# Photo Irradiation-Induced Core Crosslinked Poly(ethylene glycol)-*block*-poly(aspartic acid) Micelles: Optimization of Block Copolymer Synthesis and Characterization of Core Crosslinked Micelles

**DOI:** 10.3390/polym9120710

**Published:** 2017-12-14

**Authors:** Kouichi Shiraishi, Shin-ichi Yusa, Masanori Ito, Keita Nakai, Masayuki Yokoyama

**Affiliations:** 1 Medical Engineering Laboratory, Research Center for Medical Sciences, The Jikei University School of Medicine, 163-1, Kashiwashita, Kashiwa, Chiba 277-0004, Japan; kshiraishi@jikei.ac.jp; 2 Department of Applied Chemistry, Graduate School of Engineering, University of Hyogo, 2167 Shosha, Himeji, Hyogo 671-2280, Japan; yusa@eng.u-hyogo.ac.jp (S.-i.Y.); Hatoyaito1@gmail.com (M.I.); good.west.56@hotmail.co.jp (K.N.)

**Keywords:** polymeric micelle, photo irradiation, core crosslinking, immunogenicity of PEG

## Abstract

We used photo irradiation to design core crosslinked polymeric micelles whose only significant physico-chemical change was in their physico-chemical stability, which helps elucidate poly(ethylene glycol) (PEG)-related immunogenicity. Synthetic routes and compositions of PEG-*b*-poly(aspartic acid) block copolymers were optimized with the control of *n*-alkyl chain length and photo-sensitive chalcone moieties. The conjugation ratio between *n*-alkyl chain and the chalcone moieties was controlled, and upon the mild photo irradiation of polymeric micelles, permanent crosslink proceeded in the micelle cores. In the optimized condition, the core crosslinked (CCL) micelles exhibited no dissociation while the non-CCL micelles exhibited dissociation. These results indicate that the photo-crosslinking reactions in the inner core were successful. A gel-permeation chromatography (GPC) measurement revealed a difference between the micellar-formation stability of CCL micelles and that of the non-CCL micelles. GPC experiments revealed that the CCL micelles were more stable than the non-CCL micelles. Our research also revealed that photo-crosslinking reactions did not change the core property for drug encapsulation. In conclusion, the prepared CCL micelles exhibited the same diameter, the same formula, and the same inner-core properties for drug encapsulation as did the non-CCL micelles. Moreover, the CCL micelles exhibited non-dissociable micelle formation, while the non-CCL micelles exhibited dissociation into single block copolymers.

## 1. Introduction

Nanoparticles, such as liposomes and polymeric micelles, have been explored as drug carriers for systemic chemotherapy in recent decades [1,2,3]. In drug targeting, nanoparticles can accumulate in solid tumor tissues through the enhanced permeability and retention (EPR) effect [4,5]. A long blood circulation time-period of the nanoparticles is a prerequisite for EPR-effect-based drug targeting. To achieve the long blood circulation, nanoparticles must evade interactions with plasma proteins and monocytes (such as macrophages and liver Kupffer cells) in blood. This evasion is obtained through surface coating with a hydrophilic layer, commonly poly(ethylene glycol) (PEG) [6,7], which is a water-soluble, non-toxic polymer of very low immunogenicity and which has many uses in pharmaceutics, cosmetics, and food. This PEG surface coating is generally called PEGylation. Most PEGylated nanocarriers for therapeutic or diagnostic purposes exhibit long-circulation characteristics in blood [8,9,10,11,12,13,14,15]. Among those nanoparticles, amphiphilic block copolymer micelles (polymeric micelles) have attracted substantial attention owing to their high potential for anticancer drug targeting [1,3]. Polymeric micelles tend to have relatively small hydrodynamic diameters (10–100 nm) and to exhibit stable micelle formation above the critical micelle concentration (CMC). We examined core-shell structures of polymeric micelles by means of small angle X-ray scattering (SAXS), and SAXS measurements revealed the precise hydrophobic core size of polymeric micelles [16,17]. Polymeric micelles’ very small hydrophobic cores can encapsulate poorly water-soluble and water-insoluble drugs. Owing to the drug-encapsulation ability of polymeric micelles, clinical trials are now examining polymeric micelles loaded with anticancer drugs [18,19,20].

Polymeric micelles have shown great potential for drug targeting in terms of tumor accumulation. On the other hand, most injected polymeric micelles are excreted from blood mainly through renal filtration and spleen/liver capturing after systemic injection. A recent topic of PEGylated nanoparticles for drug targeting is the antibody responses of those PEGylated nanoparticles. Systemic injection of PEGylated nanoparticles induced the secretion of anti-PEG IgM antibodies (anti-PEG IgM), in turn changing the long blood-circulation characteristic of PEGylated nanoparticles. This phenomenon is called the accelerated blood clearance (ABC) phenomenon, which researchers have observed in mice injected with PEG-liposomes and polymeric micelles [21,22,23,24,25].

We have found that poly(ethylene glycol) chains do not strongly induce antibody responses, whereas poly(ethylene glycol) chains conjugating hydrophobic blocks become immunogenic for antibody responses [26]. Furthermore, poly(ethylene glycol) chains do not strongly bind to anti-PEG IgM, whereas poly(ethylene glycol) chains conjugating hydrophobic blocks can strongly bind to anti-PEG IgM. By contrast, PEGylation onto hydrophobic cores suppresses the binding of anti-PEG IgM to PEGylated nanoparticles. These results indicate the importance of core-forming hydrophobic blocks of hydrophilic (PEG)-hydrophobic block copolymers for anti-PEG IgM responses. In fact, even when the same PEG chain length (molecular weight = 5200) is used for polymeric-micelle formation, polymeric micelles with a 76-nm diameter induce the ABC phenomenon more effectively than polymeric micelles with a 34-nm diameter. This result implies that the size of polymeric micelles is an important factor for the ABC phenomenon.

We have uncovered evidence that PEG-block copolymers possessing hydrophobic blocks induce anti-PEG IgM responses, and that the hydrophobic block significantly enhances the immunogenicity of PEG chains. However, not only the size but also the state of block copolymers may affect the anti-PEG IgM responses related to hydrophobic blocks. Specifically, the ABC phenomenon may hinge on whether block copolymers have either a micellar form or a dissociated single polymer chain. As stated above, we have found that the larger a polymeric micelle is, the stronger the ABC phenomenon induction is. However, strong induction may result from the relative stability of large micelles. Therefore, polymeric micelles possessing discrete states are favored for elucidation of the ABC phenomenon.

One possible way to accurately investigate the anti-PEG IgM response is to compare non-dissociable polymeric micelles with dissociable polymeric micelles. The non-dissociable polymeric micelles are, as the name implies, free from dissociation; therefore, non-dissociable polymeric micelles may, in the micelle state, possess an induction mechanism of anti-PEG IgM responses. For the purposes of stable micellar carrier systems in drug delivery, inner core [27,28,29,30,31,32,33,34,35,36] and shell [37,38] crosslinking for non-dissociable micellar systems have been particularly attractive in recent years owing to the structural integrity of permanent covalent chemical bonding. In the current study, we focus on the core crosslinking of polymeric micelles, and the purpose of the study is to prepare photo-crosslinking polymeric micelles for evaluation of PEG-related immunogenicity. This approach can help reveal the in vivo behaviors, as well as the drug-release behaviors, of polymeric micelles in drug-delivery contexts.

Regarding efforts to achieve core crosslinking, there are synthetic limitations in the use of crosslinking agents. For example, a water-soluble crosslinking agent may have difficulty infiltrating a hydrophobic inner core to react with substrates. A water-insoluble agent might not be usable in aqueous media where micelles are formed. More important, crosslinking agents will change the chemical formula of block polymers, and this change might significantly affect PEG-related immune responses in vivo. We have chosen a photoreaction for the crosslinking of the polymeric-micelle cores. In the past decade, many research groups have developed various core crosslinked (CCL) approaches to enhance the stability of polymeric micelles for drug delivery. Studies have reported how they prepared photo-crosslinked micelles by using the photo-irradiated radical polymerization of poly(acrylate)s [27,28,29,30], the dimerization of cinnamoyl groups [31,32,33], and the dimerization of coumarin groups [35,36].

Herein, we report a successful development of CCL micelles by means of photo-irradiation. We used hydrocarbon chains for polymeric-micelle formation owing to their hydrophobic interactions and chalcone moieties for a photo-reactive species [39,40]. This study addresses the optimization of photo-crosslinkable block copolymer synthesis, photo-crosslinking of polymeric micelles in mild conditions, and the characterization of prepared CCL micelles. We succeeded in photo-crosslinking the inner core of polymeric micelles, and the obtained polymeric micelles exhibited high stability owing to their structural integrity.

## 2. Materials and Methods

### 2.1. Materials

α-Methoxy-ω-aminopropyl-poly(ethylene glycol) (PEG-NH_2_, *M*_w_ = 5200) was purchased from NOF Corporation (Tokyo, Japan). PEG-NH_2_ (*M*_w_ = 5200) was lyophilized from benzene before use. Sodium hydroxide, 6 N hydrochloric acid, trifluoroacetic acid (TFA), and 1,8-diazabicyclo[5.4.0]undec-7-ene (DBU) were purchased from Wako Pure Chemical Industries (Osaka, Japan). 3-Bromopropionyl chloride, 6-bromohexanoyl chloride, 9-bromononanoic acid, 1-iodopentane, and 1-iodononane, were purchased from Tokyo Kasei (Tokyo, Japan). Deuterium solvents were purchased from Sigma-Aldrich (Tokyo, Japan). Dehydrated *N*,*N*-dimethylformamide (DMF), dehydrated dimethylsulfoxide (DMSO), dehydrated dichloromethane, and thionyl chloride were purchased from Kanto Chemicals Co. (Tokyo, Japan). Dulbecco’s PBS (D-PBS(-)) was purchased from Nakarai Tesque (Kyoto, Japan). We used all these commercial reagents as purchased, unless indicated otherwise. A dialysis membrane (Spectra/Por 6, molecular weight cut off (MWCO) = 1000) was purchased from Spectrum Laboratories (Tokyo, Japan).

### 2.2. Measurements

^1^H NMR spectra were recorded on an Agilent Technologies, UNITY INOVA 400 MHz NMR spectrometer (Palo Alto, CA, USA). For ^1^H NMR measurements, block copolymers were dissolved at 10.0 mg/mL in DMSO-*d*_6_ containing 3 *v*/*v* % TFA. GPC measurements of the polymeric micelles (1.0 mg/mL in H_2_O) were carried out with an HPLC system (LC 2000 series, Jasco, Tokyo, Japan) equipped with a TSK-gel G4000-PW_XL_ column and a TSK-gel guard column PW_XL_ (eluent = H_2_O, flow rate = 1.0 mg/mL, detector = refractive index (RI)) at 40 °C. GPC measurements before and after the photo-irradiation of block copolymers were carried out by the use of a Shodex RI SE-61 refractive index detector (Showa Denko K.K, Tokyo, Japan) equipped with a Shodex Asahipak GF-7M HQ column and a GF-1G 7B guard column. Methanol containing 0.1 M LiClO_4_ was used as eluent at 40 °C. The number-average molecular weight (*M*_n_) and the weight-average molecular weight (*M*_w_) were calibrated with standard PEG samples. UV-VIS spectra were recorded on either a JASCO V-550 or a JASCO V-530 spectrophotometer (JASCO, Tokyo, Japan) with a 1.0 cm path length cell. Static light scattering (SLS) and dynamic light scattering (DLS) measurements were carried out at 25 °C with a DLS-7000 instrument (Otsuka Electronics Co., Ltd., Tokyo, Japan) equipped with an ALV5000/EPP multi-t digital time correlator.

### 2.3. Synthesis of Bromo Alkylated Chalcone Amide (***1a***–***c***)

**Scheme 1:**
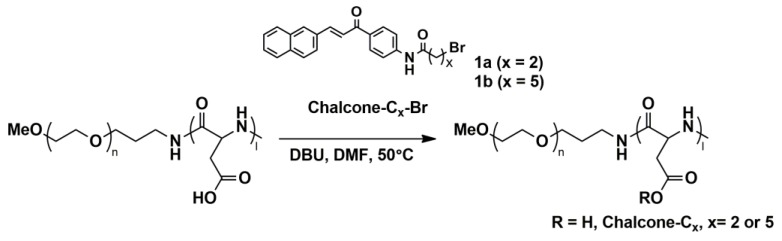
The reaction of PEG-P(Asp) with chalcone-C*_x_*-Br (Method A).

Synthesis of 1-(4-aminophenyl)-3-(2-naphthalenyl)-2-propen-1-one (4-amino-chalcone) is described elsewhere [39]. 4-amino-chalcone was stirred with an excess of 3-bromopropiponyl chloride (2–5 equivalent (eq)) in dehydrated THF at 0 °C, and the reaction mixture was gradually warmed up to r.t. After being stirred for 2 h at room temperature (r.t.), THF was removed under reduced pressure, and a yellow precipitate was obtained. The crude product was washed with diethyl ether several times. After filtration and drying under vacuum, a light-yellow powder was obtained. **1a** Yield = 77–79%. ^1^H NMR (400 MHz, CDCl_3_): δ 8.67 (d, *J* = 15.6 Hz, 2H), 8.26 (d, *J* = 8.4 Hz, 2H), 8.10 (d, *J* = 8.6 Hz, 2H), 7.92 (m, 3H), 7.71 (d, *J* = 8.6 Hz, 2H), 7.62 (d, *J* = 15.6 Hz, 2H), 7.56 (m, 4H), 7.44 (br, NH), 3.73 (t, *J* = 6.6 Hz, 2H), 3.01 (t, *J* = 6.6 Hz, 2H). In the same manner, two additional chalcone derivatives (chalcone-C*_x_*-Br) were synthesized, as shown in Scheme 1. **1b** Yield = 77–78%. ^1^H NMR (400 MHz, CDCl_3_): δ 8.67 (d, *J* = 15.6 Hz, 2H), 8.26 (d, *J* = 8.4 Hz, 2H), 8.09 (d, *J* = 8.8 Hz, 2H), 7.92 (m, 3H), 7.70 (d, *J* = 8.4 Hz, 2H), 7.63 (d, *J* = 15.2 Hz, 2H), 7.57 (m, 4H), 7.32 (br, NH), 3.44 (t, *J* = 6.8 Hz, 2H), 2.44 (t, *J* = 7.2 Hz, 2H), 1.93 (m, 2H), 1.80 (m, 2H), 0.88 (t, *J* = 6.4 Hz, 3H). **1c** Yield = 81–85%. ^1^H NMR (400 MHz, CDCl_3_): δ 8.67 (d, *J* = 15.6 Hz, 2H), 8.26 (d, *J* = 8.4 Hz, 2H), 8.09 (d, *J* = 8.8 Hz, 2H), 7.92 (m, 3H), 7.70 (d, *J* = 8.8 Hz, 2H), 7.63 (d, *J* = 15.6 Hz, 2H), 7.56 (m, 4H), 7.34 (br, NH), 3.41 (t, *J* = 6.8 Hz, 2H), 2.41 (t, *J* = 7.2 Hz, 2H), 1.85 (m, 2H), 1.76 (m, 2H), 1.48–1.34 (m, 8H) (see Figure S1a–c of the Supplementary Materials).

### 2.4. Esterification of PEG-b-P(Asp)

Synthesis of poly(ethylene glycol)-*b*-poly(aspartic acid), PEG-*b*-P(Asp) was reported in our previous report [17]. We synthesized poly(ethylene glycol)-*b*-poly(β-benzyl l-aspartate) (PEG-PBLA) by means of the ring-opening polymerization of β-benzyl l-aspartate *N*-carboxy anhydride (BLA-NCA) from α-methyl-ω-aminopropoxy poly(ethylene glycol). PEG-*b*-P(Asp) was obtained by alkaline (2.0 eq) hydrolysis of PEG-PBLA (see Figure S1d of the Supplementary Materials), followed by a hydrochloric acid treatment (2.5 eq) resulting in a free carboxylic acid form. For the esterification of PEG-*b*-P(Asp), PEG-*b*-P(Asp) was dissolved in dehydrated DMF, and a halogenated compound was added to the solution. DBU (1.05–1.20 eq/mol vs. mol of Asp) was added to the mixture, and the mixture was stirred overnight at 50 °C (16–20 h). The reaction mixture was dropped into a 10-fold volume of diethyl ether at 0 °C. The obtained precipitate was dissolved in DMSO, and hydrochloric acid (6N, 2.0 eq/mol vs. mol of DBU) was added to the solution. The resulting solution was dialyzed against H_2_O (MWCO = 1000), and lyophilized. A ^1^H NMR measurement (in DMSO-*d*_6_ containing 3 *v*/*v* % TFA) was performed for characterization of the obtained block copolymers.

### 2.5. Micelle Preparation

The obtained block copolymer was dissolved in THF, which was evaporated at 40 °C under a dry N_2_ flow. The obtained polymer film was further dried under reduced pressure for 1 h, and the film was sonicated with a VCX-750 sonicator (Sonic & Materials, Newtown, CT, USA) equipped with a 5-mm-diameter microtip in D-PBS(-). The obtained polymeric-micelle solutions were centrifuged (3900 rpm, 10 min) for removal of possible insoluble precipitates. We used a filter unit equipped with an Amicon Ultra-15 (*M*_WCO_ = 100 k) to concentrate the polymeric-micelle solutions, which were passed through a 0.22 μm poly(vinylidene difluoride) (PVDF) filter unit (Nihon Millipore, K.K., Tokyo, Japan).

### 2.6. Photo-Crosslinking of Polymeric Micelles

Photo irradiation was applied to polymeric micelles at 5.0 mg/mL in a normal saline (N.S.) solution using an Asahi Spectra MAX-300 (Asahi Spectra Co., Ltd., Tokyo, Japan) equipped with a 300-W Xe-lamp and a 275-nm cutoff filter at 25 °C. The intensity was set to 8.0 mW/cm^2^ at 350 nm for 3 h. For confirmation of the reaction’s progress, a UV-VIS spectrum of the polymeric-micelle solution was monitored until an absorption peak at 350 nm disappeared.

### 2.7. GPC Elution Measurements of Polymeric Micelles

At a concentration range between 5.0 and 1.0 mg/mL, the CCL micelles and the non-CCL micelles (100 μL) were injected into the GPC, and the obtained peak area was normalized (μVsec/injected weight of polymeric micelles) in relation to either the CCL or the non-CCL micelles. We measured whole peak areas of both the CCL micelles and the non-CCL micelles by comparing the peak areas of micelles with one another upon injection of the micelles into the HPLC without the column (Figure S2 in the Supplementary Materials).

### 2.8. Preparation of Adriamycin-Encapsulated Polymeric Micelles

Block copolymers were dissolved in DMF, and Adriamycin (ADR) was dissolved in DMF containing trimethylamine (1.2 molar equivalent). The two solutions were mixed and dialyzed against H_2_O by the use of a dialysis membrane with MWCO = 1000 (Spectra/Por 6). The obtained polymeric micelle solutions were centrifuged (3900 rpm, 10 min) for removal of possible aggregates. We used a filter unit equipped with an Amicon Ultra-15 (MWCO = 100,000) to concentrate the polymeric-micelle solutions. The obtained polymeric-micelle solutions were passed through a 0.22-μm PVDF filter unit.

### 2.9. Drug-Release Experiment

ADR-encapsulated CCL micelles and ADR-encapsulated non-CCL micelles were filled in a dialysis unit, with each micelle solution (100 μL) containing 13 μg ADR (Slide-A-Lyzer MINI Dialysis Unit (MWCO = 3500), Thermo Scientific, Japan), and were dialyzed against a 40-fold volume of either a 0.1-M phosphate buffer (pH = 7.4) or a 0.1-M acetate buffer (pH = 5.0) with stirring at r.t., respectively. An aliquot of the dialysate was freeze-dried, and its ADR content was determined by means of reverse-phased HPLC equipped with a μ-Bondasphere column (Waters, C4 bonded phase endcapped with 5-μm silica, pore size = 100 Å, eluent = acetonitrile containing 1% acetic acid, flow rate = 1.0 mg/mL, detector = UV at 485 nm) at 40 °C. This drug-release measurement was performed in triplicate.

## 3. Results and Discussion

### 3.1. Optimization of Photoreactive Chalcone-Conjugated Block Copolymers

Introduction of hydrophobic moieties into PEG-*b*-P(Asp) leads to the formation of polymeric micelles in aqueous media [16,17]. We selected *n*-alkyl chains, such as *n*-pentyl or *n*-nonyl, as hydrophobic moieties [24,25]. Together with these alkyl chains, we used alkylated photo-crosslinkable chalcone derivatives as a photo sensitive compound. We examined the following three methods for optimization of the conjugation reaction.

#### 3.1.1. Method A

**Chart 1:**
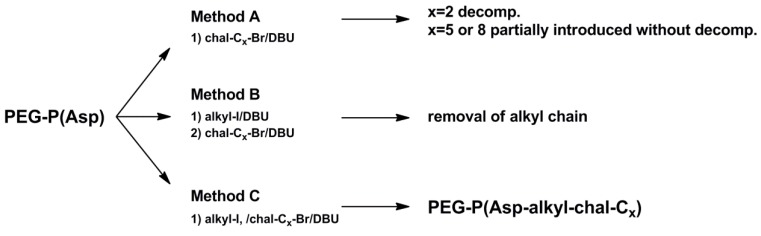
Summary of PEG-P(Asp-alkyl-chal-*C_x_*) synthesis.

In our first examination, we performed esterification of PEG-*b*-P(Asp) with a chalcone derivative having an ethylene spacer, chal-C_2_-Br (**1a**), at 50 °C in dehydrated DMF (see Scheme 1, method A in Chart 1). However, we found that a very small number of chal-C_2_ were conjugated to PEG-*b*-P(Asp) chains, and absorption at 350 nm, which corresponds to the π–π* transition state of chalcone moieties [39], was decreased after the reaction (see Figure S3 of the Supplementary Materials). This decrease indicates that a side reaction, which changed an absorption spectrum of chal-C_2_, occurred during the reaction. We confirmed that mixtures of **1a** with PEG-*b*-P(Asp) (without DBU) or **1a** with DBU (without PEG-*b*-P(Asp)) in the same reaction condition did not show a change in the π–π* band (data not shown). The conjugation reaction occurred between carboxylate anion and alkyl halides in polar DMF. In this condition, the reactivity of alkyl halides in **1a** (in this case Br–CH_2_CH_2_–C(O)) was low owing to an electron-withdrawing carbonyl group attached to the ethylene group. However, reactive carboxylate anions remained in the mixture, and might have reacted with chalcone moieties instead of alkyl halides. We examined a reaction involving PEG-*b*-P(Asp) and 3-bromo-*N*-butyl-propanamide (BrCH_2_CH_2_C(O)NH*n*C_4_H_9_) (3-BNBPA), which is similar in chemical structure to **1a**, in the reaction condition identical to those of chal-C_2_-Br and PEG-*b*-P(Asp) (2.0 eq/mol 3-BNBPA and 1.2 eq/mol DBU were applied; see Table S1 of the Supplementary Materials). This reaction yielded only 24% esterification for PEG-*b*-P(Asp) (see Figure S1e of the Supplementary Materials). From this result, we concluded that less reactive alkyl halides led to a smaller incidence of 3-BNBPA conjugation to PEG-*b*-P(Asp), and that the remaining carboxylate anions attacked chalcone π-conjugated moieties. Owing to this unfavorable change in the chalcone π-conjugated moieties in which a photo crosslinked reaction had to occur, we used long alkylated chalcone derivatives, such as pentyl spacers (chal-C_5_-Br, **1b**) and octyl spacers (chal-C_8_-Br, **1c**), instead of **1a**. These long alkylated chalcone derivatives were revealed to possess sufficient reactivity with carboxylate anions. Although the number of introduced chal-C_5_ derivatives was not large enough (the number of introduced chalcone derivatives = 1.7, yield from feed mol was 22%; see Table S2 of the Supplementary Materials), we found that chal-C_5_ was successfully conjugated into PEG-*b*-P(Asp) without a significant change in chalcone derivative’s absorption band. The fact that methylene chains are, in general, longer than pentyl chains (*n* > C_5_) in chalcone derivatives significantly reduced the change in chalcone derivatives’ absorption peak in UV-VIS spectroscopy.

#### 3.1.2. Method B

**Scheme 2:**
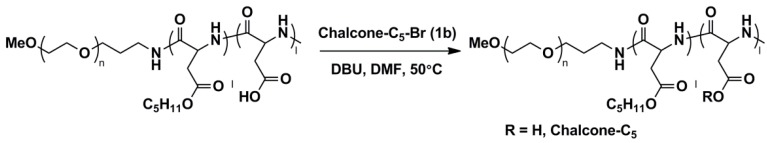
The reaction of PEG-P(Asp-pentyl) with chalcone-C*_x_*-Br (Method B).

We performed two-step reactions as shown in part B of Chart 1. We conjugated pentyl groups to PEG-*b*-P(Asp) in the first esterification step (see Figure S1f of the Supplementary Materials), followed by conjugation of **1b** in the second step (see Scheme 2). The reaction of **1b** proceeded with PEG-P(Asp(26)-pentyl(76)), whose aspartic acid residues had been conjugated with pentyl groups (76% of the pentyl group in 26 aspartic-acid residues), under the same reaction condition as the first esterification step (see Scheme 2). The obtained block copolymer did not exhibit the change in chalcone derivatives’ absorption peak, and we controlled the number of conjugated chal-C_5_ by controlling the **1b** feed amount, as shown in Table 1. However, we found a decreasing number of pentyl groups with an increasing number of conjugated chal-C_5_ in the PEG-P(Asp-pentyl). This result indicates that chal-C_5_ conjugation took place with removal of the pentyl group.

#### 3.1.3. Method C

**Scheme 3:**
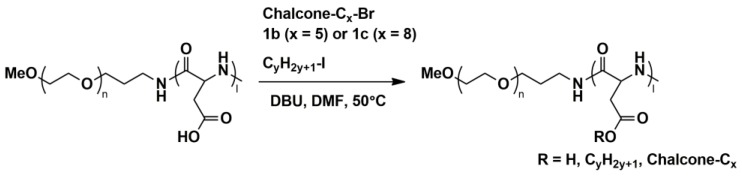
The reaction of PEG-P(Asp) with chalcone-C*_x_*-Br (Method C).

**Figure 1:**
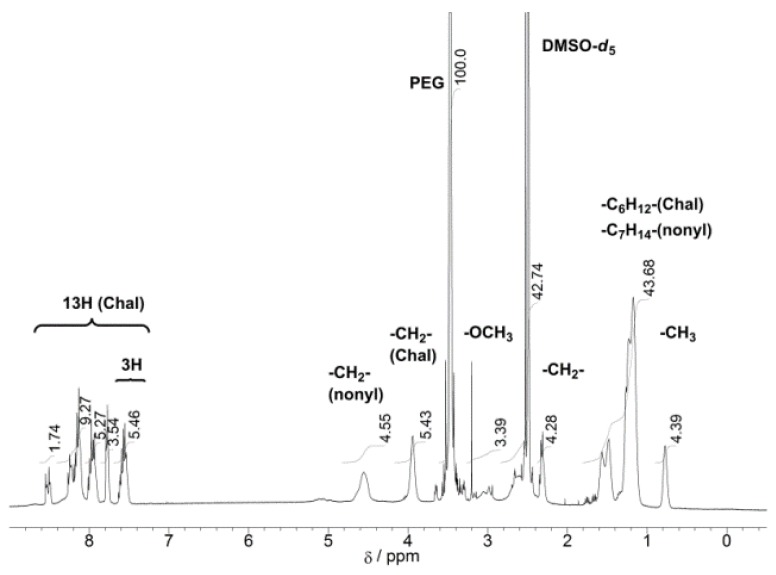
^1^H NMR spectrum of the PEG-P(Asp-nonyl-chal-C_8_) block copolymer in DMSO-*d*_6_ containing 3 *v*/*v* % TFA.

As the two-step reactions did not successfully provide a favorable composition, we performed an experiment involving a reaction of PEG-*b*-P(Asp) with alkyl halides and chal-C*_x_*-Br (*x* = 5 or 8) simultaneously (see Scheme 3, method C in Chart 1). ^1^H NMR was used for characterizations of PEG-P(Asp-alkyl-chal-C*_x_*), as shown in Figure 1. We calculated the number of conjugated alkyl chains and chal-C_x_ derivatives by comparing the integration among the PEG’s methylene protons (degree of polymerization = 119), the terminal methyl protons of the conjugated alkyl chains at 0.77 ppm, and 3H aromatic protons of chal-C*_x_* derivatives at 7.58 ppm.

The reaction proceeded well without a change in absorption peak at 350 nm. As shown in Table 2 (Run 1–3), the obtained number of pentyl groups and chal-C_5_ derivatives was well controlled by the feed ratio between 1-iodopentane and **1b**. In contrast, the chalcone having a C_5_ alkyl chain (**1b**) conjugation reaction did not proceed in the mixture with *n*-C_9_H_19_-I (nonyl iodide), while conjugation of nonyl iodide took place for the block copolymer (see Run 4 in Table 2). The results indicate that the reaction of nonyl iodides to PEG-*b*-P(Asp) was faster than the reaction of **1b** to PEG-*b*-P(Asp). Once the nonyl chain was conjugated into PEG-*b*-P(Asp), a short pentyl chain having a bulky chalcone moiety possibly could not reach the aspartic acid residues at the PEG-*b*-P(Asp) backbone. From the above-mentioned results, we found that at least an equivalent number (*x* = *y* − 1) of the methylene groups was necessary for the conjugation of chalcone derivatives to obtain PEG-P(Asp-C_y_H_2y+1_-chal-C_x_) and long alkyl chains, such as a nonyl chain, exhibit better reactivity. As we expected, the reaction between PEG-P(Asp), *n*-C_9_H_19_-I, and a C_8_ alkyl chain (**1c**) yielded good results (Table 2, Run 5–8). Results indicated that reaction efficiencies (% *N*(found)/*N*(feed)) of each substrate was 33–42% for **1c** and 28–47% for *n*-C_9_H_19_-I, respectively, therefore, we succeeded in preparing PEG-P(Asp-C*_y_*H_2*y*+1_-chal-C*_x_*) in a well-controlled manner.

### 3.2. Photo-Crosslinking of Polymeric Micelles

We used a solvent evaporation–sonication method to prepare PEG-P(Asp-nonyl-chal-C_8_) micelles [16,17]. In addition, we performed DLS and UV-VIS measurements to confirm the obtained PEG-P(Asp-nonyl-chal-C_8_) micelles’ characteristics. As shown in Table 3, diameters of the micelles were in a range of 20–40 nm, except in Run 1. The block copolymers having 18.2 nonyl chains and 2.8 Chal-C_8_ groups exhibited a mixture of micelle and vesicle formation, which we determined by means of TEM (see Figure S4 of the Supplementary Materials). This helped clarify why we observed this exceptional diameter in Run 1 of Table 3.

**Figure 2:**
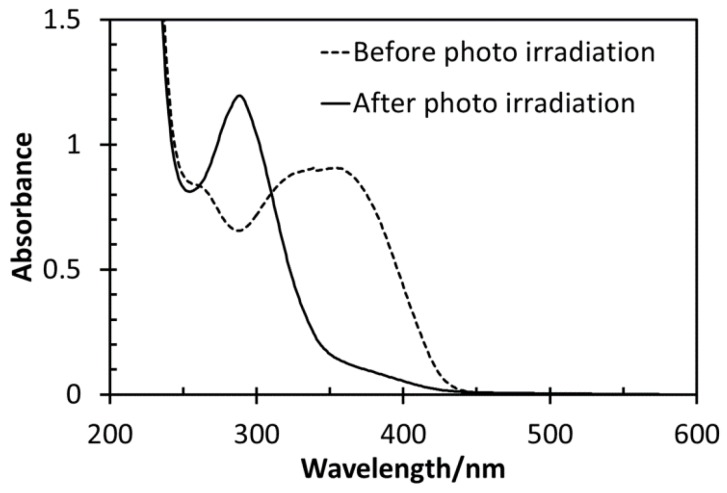
UV-VIS absorption spectra of PEG-P(Asp-nonyl-chal-C_8_) (nonyl = 7.0, chal-C_8_ = 8.6) micelles before photo irradiation (solid line) and after photo irradiation (dashed line) in a 0.1 mg/mL solution.

The obtained micelles exhibited a maximum π–π* absorption peak at 350 nm. We performed photo-irradiation on the micelle concentration at 5.0 mg/mL with an intensity of 8.0 mW/cm^2^ at 350 nm of UV-VIS light for 3 h. In this mild light-intensity condition, no absorption peak around 325–360 nm was found after 3 h, and a new absorption peak at 300 nm appeared upon photo-irradiation, as shown in Figure 2. This spectrum change was due to single bond formation via [2 + 2] addition of the chalcone moieties (see Scheme S1 of the Supplementary Materials). The disappearance of the peak in the range extending from about 325 to 360 nm indicates that the photo reaction proceeded in polymeric micelles’ inner cores. In other photo-crosslinking systems, many studies have applied high light intensities at shorter wavelengths to polymeric micelles [31,32,33,34,35,36]; however, in the current study, we have successfully performed a crosslinking reaction in polymeric micelles’ core with photo irradiation at mild light intensity.

### 3.3. Measurement of Size and Molecular Weight upon Photo-Irradiation

We used GPC measurements in an organic solvent to compare the molecular weight of PEG-P(Asp-nonyl-Chal-C_8_) block copolymers before and after photo irradiation. The CCL micelles exhibited an *M*_n_ of 1.6 × 10^5^ with a *M*_w_/*M*_n_ of 1.63, while the non-crosslinked block copolymer exhibited an *M*_n_ of 5.1 × 10^3^ with a *M*_w_/*M*_n_ of 1.29 (Run 4 of Table 3) in GPC (eluent = methanol containing 0.1 M LiClO_4_) (see Figure S5 of the Supplementary Materials). The obtained *M*_n_ of non-crosslinked block copolymers was in good agreement with the ^1^H NMR values, indicating that the non-crosslinked block copolymers did not form aggregates in methanol.

It should be noted that the *M*_n_ values of PEG-P(Asp-nonyl-Chal-C_8_) block copolymers (nonyl = 7.0, chal-C_8_ = 8.6) and the CCL micelles in DMF were 5.6 × 10^4^ and 6.5 × 10^5^ with *M*_w_/*M*_n_ = 1.14 and 1.17, respectively (data not shown). The molecular weight of the PEG-P(Asp-nonyl-Chal-C_8_) block copolymers, when measured in DMF, was greater than the corresponding molecular weight obtained in the context of ^1^H NMR. This fact indicates that PEG-P(Asp-nonyl-Chal-C_8_) block copolymers underwent aggregation before photo-crosslinking in DMF. Therefore, an appropriate choice of solvents is required for determining the actual molecular weight of PEG-P(Asp-nonyl-Chal-C_8_) block copolymers in GPC. We have succeeded in preparing CCL micelles from PEG-P(Asp-nonyl-chal-C_8_) block copolymers (nonyl = 7.0, chal-C_8_ = 6.9; nonyl = 7.0, chal-C_8_ = 8.6; Runs 3 and 4 of Table 3). By contrast, when in methanol, PEG-P(Asp-nonyl-chal-C_8_) block copolymers (nonyl = 9.3, chal-C_8_ = 5.6; Runs 2 of Table 3) exhibited several peaks on the GPC chart because the number of chalcone derivatives that had undergone incomplete photo-crosslinking was smaller than the number of chalcone derivatives that had undergone complete photo-crosslinking. The number of chalcone derivatives in the polymeric micelles’ core was revealed to be an important factor in our effort to complete the photo-crosslinking. To confirm the formation of CCL micelles (nonyl = 7.0, chal-C_8_ = 8.6), we characterized the obtained polymeric micelles by means of DLS and SLS.

**Figure 3:**
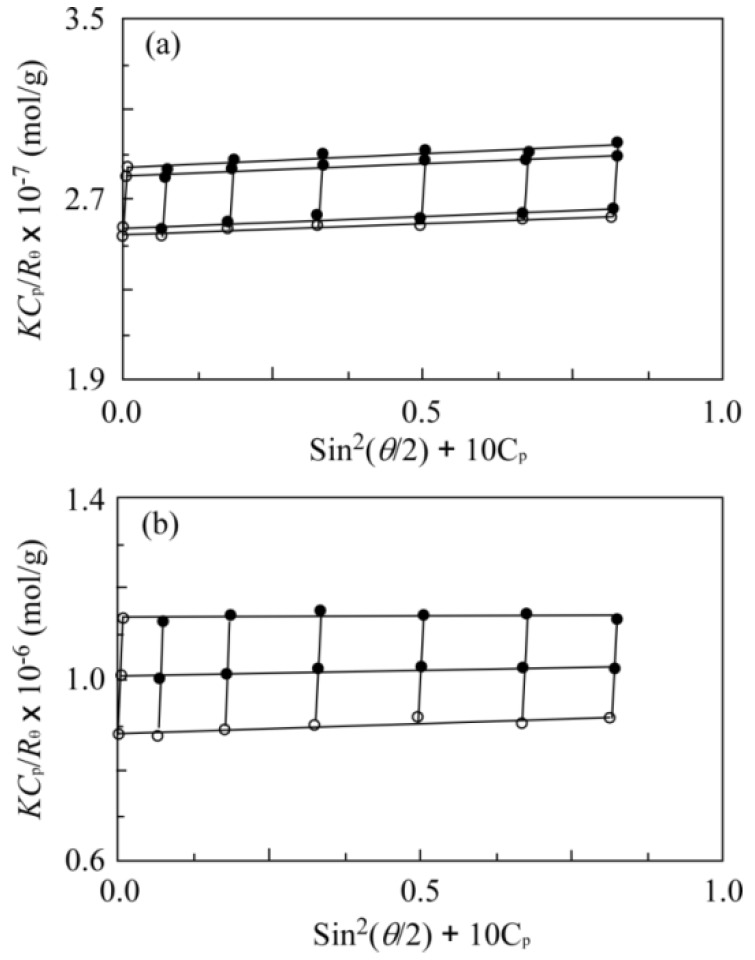
Zimm plots of PEG-P(Asp-nonyl-chal-C_8_) (nonyl = 7.0, chal-C_8_ = 8.6) micelles (**a**) before and (**b**) after photo-crosslinking; and (**a**) in normal saline and (**b**) in methanol containing 0.1 M LiClO_4_.

Our DLS measurements yielded polymeric micelles whose diameters after photo-irradiation were nearly the same as the diameters before photo-irradiation in aqueous media (Table 4). This result indicates that photo-irradiation did not affect the radius of polymeric micelles in aqueous media. Figure 3 presents the Zimm plots for the CCL-micelles obtained by means of SLS measurements either in normal saline before the photo-crosslinking or in methanol containing 0.1 M LiCO_4_ after the photo-crosslinking. Table 4 shows the SLS data and hydrodynamic radius (*R*_h_) for the polymeric micelles. We could not obtain SLS data regarding PEG-P(Asp-nonyl-chal-C_8_) block copolymers in methanol owing to the very weak scattering intensities of the unimer form of the block copolymers. To estimate the values of apparent *M*_w_ for polymeric micelles before and after photo-crosslinking, we extrapolated the polymer concentration (*C*_p_) and scattering angle (θ) to zero, and to estimate the values of the radius of gyration (*R*_g_) and the second virial coefficient (*A*_2_), we referred respectively to the slope of the angular dependence and the slope of the concentration dependence in the Zimm plots. Polymeric micelles’ *M*_w_ before photo-crosslinking was 3.93 × 10^6^, and the aggregation number (*N*_agg_) was 310, as estimated by the molecular weight of the block copolymer. After photo-crosslinking, we dialyzed the CCL-micelle solution against methanol to exchange the solvent and remove possible non-crosslinked block copolymers. The *M*_w_ of CCL micelles was 1.14 × 10^6^, and the *N*_agg_ was 90. The *R*_g_/*R*_h_ values of the polymeric micelles before and after photo-crosslinking were 0.837 and 0.982, respectively. The *R*_g_/*R*_h_ value of the CCL micelles indicates that the structure of their inner core was less dense than the inner core of the non-CCL micelles in methanol.

### 3.4. DLS Measurements of CCL and Non-CCL Micelles

**Figure 4:**
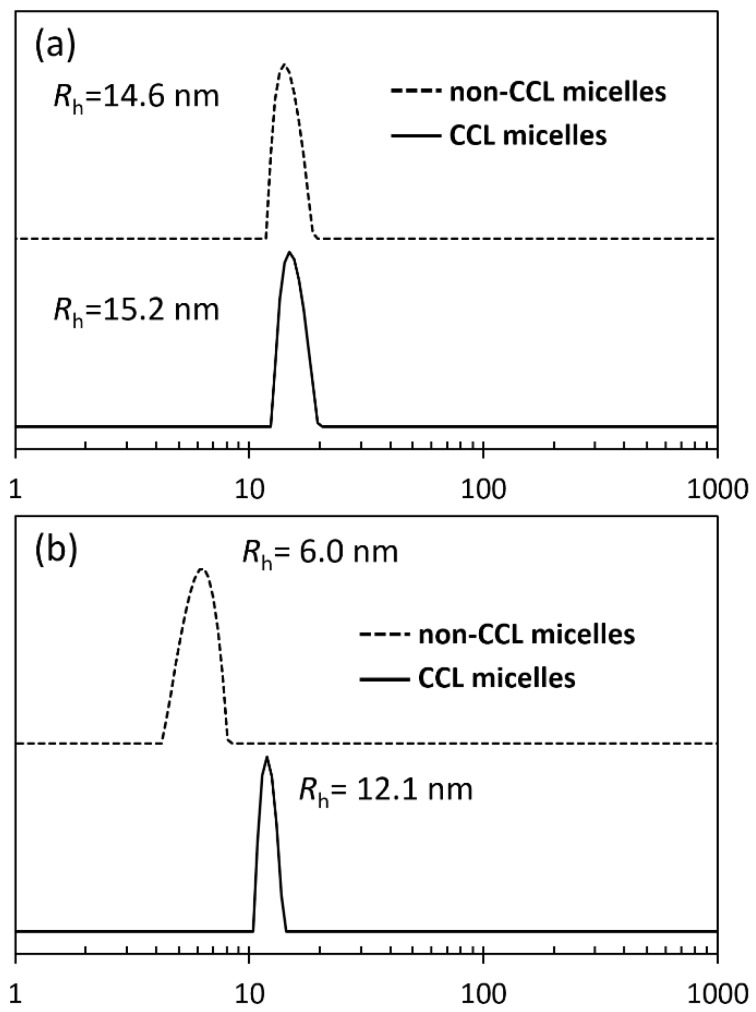
DLS charts of PEG-P(Asp-nonyl-chal-C_8_) micelles (nonyl = 7.0, chal-C_8_ = 8.6) before and after photo irradiation (**a**) in normal saline and (**b**) in methanol containing 0.1 M LiClO_4_.

Owing to the aggregation of PEG-P(Asp-nonyl-chal-C_8_) block copolymers as confirmed by GPC measurements in DMF, we dissolved the CCL micelles and block copolymers in methanol containing 0.1 M LiClO_4_. Figure 4 exhibits the radius of CCL and non-CCL micelles before and after photo irradiation. We have observed that the CCL and the non-CCL micelles exhibited the same diameters in normal saline (Figure 4a). In methanol containing 0.1 M LiClO_4_, the radius of the CCL micelles indicated the presence of an aggregate form, while the radius of the non-photo-irradiated block copolymers indicates solely the presence of a unimer form without the aggregation, as shown in Figure 4b. These findings suggest that photo-crosslinking of the cores proceeded successfully and that, consequently, the polymeric micelles did not dissociate into single block copolymers in methanol containing 0.1 M LiClO_4_.

### 3.5. GPC Measurements of CCL and Non-CCL Micelles for Calculation of CMC

Measurements of the emission and excitation spectra of pyrene encapsulated in polymeric micelles provided critical micelle concentration (CMC) values of polymeric micelles [41,42]. We followed a pyrene-encapsulation method to determine the CMC values of the CCL and the non-CCL micelles (nonyl = 7.0, chal-C_8_ = 8.6) [42]; however, emission spectra of the pyrene encapsulated polymeric micelles exhibited a new broad absorption peak at 480 nm in a concentration range extending from 0.1 to 2.0 mg/mL. This peak resulted from exciplex formation between pyrene and chalcone moieties in the inner core, since PEG-P(Asp-nonyl) micelles (without chalcone moieties) did not exhibit such a new peak. Owing to the exciplex formation between pyrene and chalcone moieties, we could not estimate the CMC values of the two types of polymeric micelles (see Figure S6 of the Supplementary Materials).

**Figure 5:**
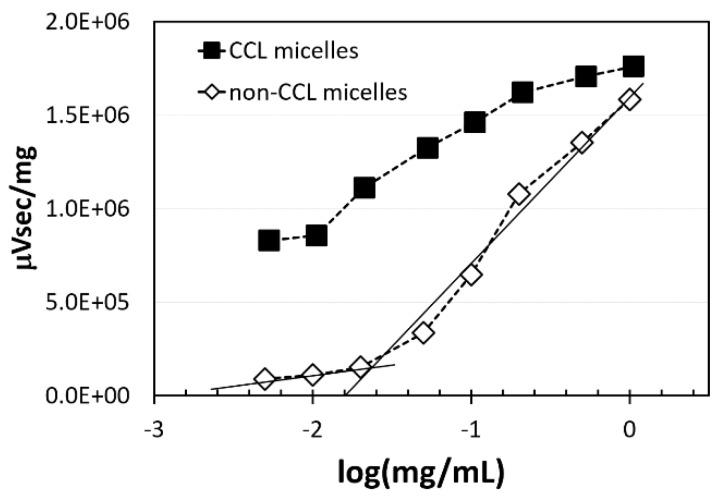
GPC measurements conducted before (diamond) and after (square) the photo-crosslinking of polymeric micelles. Concentrations of the polymers were in a range extending from 0.5 μg/mL to 1.0 mg/mL. Y axis indicates normalized elution-peak areas per injected weight gram of polymeric micelles in GPC measurements. The obtained normalized elution-peak area was plotted.

Rather than measure the emission and excitation spectra of pyrene, we measured concentration-dependent GPC elution peaks in order to observe the shear stress-mediated dissociation and adsorption behaviors of the CCL and the non-CCL micelles (nonyl = 7.0, chal-C_8_ = 8.6). We have proved that this concentration-dependent GPC measurement deeply correlated with the stability of micelles in vivo [17]. Figure 5 exhibited plots of the normalized elution-peak areas (μVsec/injected weight of polymeric micelles) of the CCL and the non-CCL micelles at each concentration. The plots of non-CCL micelles dropped at a higher concentration than did the plots of CCL micelles. Under this GPC condition, the non-CCL micelles exhibited almost no elution peak below 0.01 mg/mL. These two kinds of micelles were identical to one another regarding diameter and chemical formula, but exhibited different plot profiles. The difference in GPC measurements was due mainly to the adsorption—stemming from hydrophobic interactions—of either the unimer form or the polymeric micelles onto the GPC column. If the polymeric micelles exhibited no adsorption on the column, the plots of the normalized peak areas should have been flat. Owing to the hydrophobic interaction between the column and unimer forms of the block copolymers, the normalized peak area of non-CCL micelles drastically decreased in the diluted conditions. However, as shown in Figure 5, the plots of the CCL micelles gradually dropped even after photo-crosslinking. It should be noted that adsorption behaviors of those micelles depend on the number of adsorption sites in a dynamic environment (flow rate and flow pressure). Therefore, a significant amount of injected micelles adsorbed onto the GPC column, and a low concentration of micelles exhibited significant decreases in elution-peak areas, whereas a high concentration of micelles exhibited negligible decreases in elution-peak areas. In fact, 88% of the non-CCL micelles were eluted at 1.0 mg/mL, whereas 98% of the CCL micelles were eluted at the same injection concentration. Therefore, this pattern was due not to dissociation, but perhaps to the hydrophobic interactions between the CCL micelle’s inner core and the column’s surface. These data show that there was a stability difference between the CCL micelles and the non-CCL micelles in the GPC measurements.

The detection limit of the non-CCL micelles’ concentration was 0.01 mg/mL. Moreover, as shown in Figure 5, an obtained CMC value from a crossing point between the two approximation straight lines was 20 μg/mL. This CMC value was in a good agreement with the CMC of benzylated PEG-*b*-P(Asp) block copolymer micelles obtained in pyrene excitation and emission measurements (5–40 μg/mL) [16]. By contrast, the CCL micelles in the GPC measurements did not exhibit a drastic decrease in the elution-peak area. We observed the CCL micelles exhibiting high stability even in dilute conditions in the GPC experiment, as shown in Figure 5.

### 3.6. Comparisons of Two Micelles’ Adriamycin Encapsulation and Release Behaviors

In this experiment, we performed an in vitro drug-release experiment to clarify the effects of the inner core’s hydrophobic properties on the release of Adriamycin (ADR). We used two block copolymers for ADR encapsulation (in Table 2, see Run 6’s block copolymer (nonyl = 9.3 and chal-C_8_ = 5.6) and Run 7’s block copolymer (nonyl = 7.0 and chal-C_8_ = 6.9)). ADR was encapsulated in the two types of block copolymer micelles in good yields (75–80% in 2.0 wt % ADR feed to a block copolymer), and the obtained ADR-encapsulated micelles respectively had a 19-nm diameter (nonyl = 7.0 and chal-C_8_ = 6.9) and a 33-nm diameter (nonyl = 9.3 and chal-C_8_ = 5.6). We examined photo irradiation to these ADR-encapsulated polymeric micelles. It should be noted that ADR is a photo-sensitive compound, so we took additional precautions during the photo irradiation [43,44]. Upon photo irradiation, a 50% decrease in ADR absorption at 488 nm was observed in the UV-vis spectrum of the 19-nm-diameter polymeric micelles, while 72% of the ADR absorption remained for the 33-nm-diameter polymeric micelles. The smaller-diameter (19 nm) ADR-encapsulated polymeric micelles exhibited much faster ADR decomposition upon photo-irradiation than did the larger-diameter (33 nm) ADR-encapsulated polymeric micelles. Owing to the decomposition of ADR upon photo irradiation, only the 33-nm-diameter CCL or non-CCL micelles were employed for further examination in this study.

**Figure 6:**
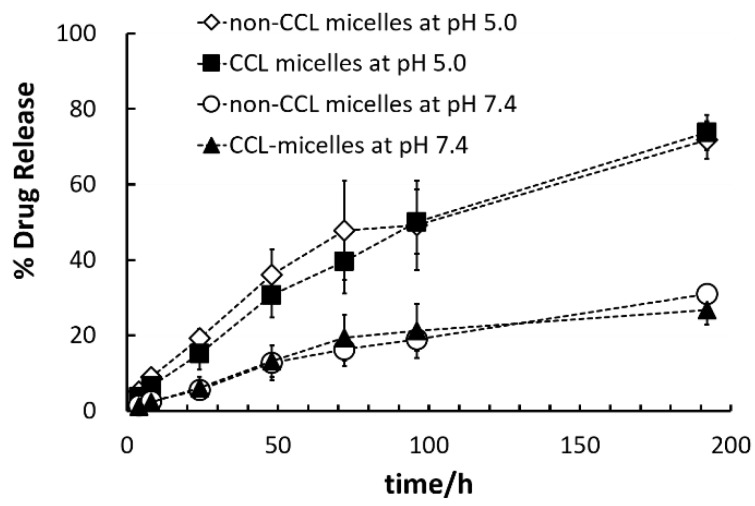
Release of Adriamycin from polymeric micelles in different media where either pH = 7.4 (d-PBS) or pH = 5.0 (acetate buffer). Diamonds and squares indicate the after and before photo-irradiation contexts, respectively. The release assay was performed in triplicate.

We examined the ADR-release behaviors of both the CCL and the non-CCL micelles in conditions either at pH = 7.4 or at pH = 5.0 by means of a dialysis method. ADR-encapsulated micelles were filled in a dialysis bag, and we monitored released-ADR in the outer medium until day 8. Results are shown in Figure 6. Both the CCL and the non-CCL micelles exhibited similar ADR-release behaviors in the two conditions. After 4 days, both the CCL micelles and the non-CCL micelles released about 20% of their ADR at pH = 7.4 and about 50% at pH 5.0. Owing to the higher solubility of ADR in the acidic medium, the two types of micelles exhibited faster ADR release at pH = 5.0 than at pH = 7.4. However, ADR was stably encapsulated in the two types of micelles, and photo irradiation did not change ADR-releasing behaviors. This was unexpected for us. We had expected that two micelles exhibit different ADR-release behaviors. In fact, polymeric micelles have emerged as a novel anticancer-drug carrier in drug-delivery systems in recent decades, research has not yet completely elucidated the drug-release mechanism involving polymeric micelles, particularly regarding whether the drug release is dominated by a mechanism of diffusion in the inner core, by a mechanism of the dissociation of polymeric micelles, or by both of these mechanisms. Results of ADR-release experiments show that a diffusion mechanism contributed to ADR release from polymeric micelles’ inner core in vitro and that a mechanism of the dissociation of polymeric micelles did not. This result was probably due to two different hydrophobic characteristics of bulky aromatic rings of ADR and long alkyl chains. Previous study shown that chemically conjugated ADR to P(Asp) helps to maintain physically entrapped ADR in micelles’ core [44]. Therefore, hydrophobic interactions between aromatic rings, as well as π–π interactions improve stability of ADR encapsulation, and these interactions may be more stable for crosslinked micelles, which neighboring hydrophobic blocks can interact easily, than the dissociable micelles. At least, the obtained results proved that photo-irradiation did not change the ADR-releasing behaviors.

## 4. Conclusions

In the current study, we succeeded in preparing two distinct types of polymeric micelles: those with photo-crosslinking and those without photo-crosslinking. We focused on photo-crosslinking the polymeric micelles’ cores so that the two types of polymeric micelles would possess the same chemical formula. We succeeded in syntheses of photo-crosslinkable block copolymers—PEG-P(Asp-alkyl-chal-C*_x_*). We found that reactions among photo-crosslinkable chalcone moieties, alkyl chains, and PEG-*b*-P(Asp) successfully proceeded when we mixed all the substrates at once. The obtained block copolymers formed polymeric micelles with diameters ranging from 20 to 40 nm in aqueous media. We optimized the composition of PEG-P(Asp-nonyl-chal-C_8_) micelles for successful photo-crosslinking in the cores. Mild photo-irradiation led to the crosslinking of chalcone moieties in the polymeric micelles’ inner cores. The core crosslinked micelles having a diameter of between 25 and 30 nm were present in both aqueous media and methanol, while the non-CCL micelles exhibited dissociation in methanol. We examined GPC experiments to evaluate dissociation and adsorption behaviors of the two types of micelles. The CCL micelles exhibited greater elution-peak areas than the non-CCL micelles owing to either the complete absence of—or the very small presence of—dissociation-related adsorption onto the GPC column. No release-profile difference emerged between the CCL and the non-CCL micelles when the encapsulated Adriamycin was released from the micelles’ inner cores. These results indicate that we obtained our desired property associated with the polymeric micelles: namely, the CCL micelles and the non-CCL micelles shared identical diameters, chemical formulas, and hydrophobic properties, even though the CCL micelles exhibited no dissociation into a unimer of block copolymers. We observed cmc of the non-CCL micelles and no cmc-related dissociation behavior of the CCL micelle. By using these two micelles, we can evaluate PEG-related immunogenicity in both forms: a micelle form and a dissociated polymer form. This is a big challenge to elucidate mechanisms of polymeric micelles’ immunogenicity in vivo.

## Figures and Tables

**Table 1:** Chal-C_5_ conjugation to PEG-P(Asp(26)-pentyl(76)).

Run	Chal-C_5_-Br Feed		DBU Feed	Chal-C_5_ Found	C_5_H_11_ Found
	Number	Ratio	/eq *	Number	Number
1	31.2	1.2	1.2	2.3	2.4
2	26.0	1.0	1.0	2.1	2.5
3	20.8	0.8	0.75	1.4	3.4
4	15.6	0.6	0.5	0.6	7.7

* eq indicates feed equivalent mol of 1,8-diazabicyclo[5.4.0]undec-7-ene (DBU) vs. 26 Asp residue in PEG-P(Asp(26)).

**Table 2:** Results of the conjugation of chal-C*_x_* and *C_y_H*_2*y*+1_ to PEG-P(Asp(26)).

Run	Chal-C*_x_*-Br Feed			C*_y_*H_2*y*+1_-I Feed			Chal-C*_x_* Found	C*_y_*H_2*y*+1_ Found
	*x*	eq *	Number	*y*	eq *	Number	Number	Number
1	5	0.3	7.8	5	1.1	28.6	**2.6**	**8.8**
2	5	0.3	7.8	5	1.5	39.0	**3.5**	**12.9**
3	5	0.3	7.8	5	2.0	52.0	**2.7**	**14.2**
4	5	0.3	7.8	9	1.5	39.0	**0.0**	**13.2**
5	8	0.3	7.8	9	1.5	39.0	**2.8**	**18.2**
6	8	0.6	15.6	9	1.2	31.2	**5.6**	**9.3**
7	8	0.8	20.8	9	1.0	26.0	**6.9**	**7.0**
8	8	0.8	20.8	9	0.8	20.8	**8.6**	**6.0**
9	8	0.8	20.8	9	0.6	15.6	**8.8**	**4.4**

* equivalent vs. 26 Asp residues. DBU equivalent = 1.1 vs. Asp.

**Table 3:** The number of introduced *n*-nonyl and chal-C_8_ moieties in block copolymers and the size of the prepared polymeric micelles.

Run	C_9_H_19_/N	Chal-C_8_/N	Diameter/nm
1	18.2	2.8	129 *^1^
2	9.3	5.6	37
3	7.0	6.9	17
4	7.0	8.6	29

*^1^ The diameter in run 1 was calculated by means of a cumulative method.

**Table 4:** Diameter, weight-average molecular weight (*M*_w_), second virial coefficient (*A*_2_), radius of gyration (*R*_g_), and aggregation number (*N*_agg_) of non-core crosslinked (CCL) and CCL micelles.

Run	DLS Diameter/nm	SLS *M*_w_ × 10^−6^	*A*_2_ (cm^3^·mol/g^2^)*^b^* × 10^5^	*R*_g_*/R*_h_	*N*_agg_	Media
Non-CCL micelle	29	3.93	1.55	0.837	310	in NS ^1^
CCL micelle	30	1.14	1.29	0.982	90	Methanol ^2^

^1^ normal saline, ^2^ methanol containing 0.1 M LiClO_4_. The composition of the PEG-P(Asp-nonyl-chal-C_8_) used in this study was nonyl = 7.0, chal-C_8_ = 8.6.

## Supplementary Materials

The following are available online at www.mdpi.com/2073-4360/9/12/710/s1, Additional data: Figure S1: ^1^H NMR of (a–c) chalcone derivatives, (d) ^1^H NMR of PEG-P(Asp) block copolymer, (e–f) esterified PEG-P(Asp) block copolymers. Figure S2: GPC experiments for estimating the non-adsorption behaviors of CCL micelles and non-CCL micelles. Figure S3: UV-VIS spectrum of PEG-P(Asp-chal-C_2_). Figure S4: TEM images of the prepared PEG-P(Asp-nonyl-chal-C_8_) micelles. Figure S5: GPC traces before and after photo-irradiation in MeOH containing 0.1 M LiClO_4_. Figure S6: emission spectra of pyrene-encapsulated PEG-P(Asp-nonyl-chal-C_8_) micelles before and after photo-irradiation.
