# Supplementary material for: Photo Irradiation-Induced Core Crosslinked Poly(ethylene glycol)-block-poly(aspartic acid) Micelles: Optimization of Block Copolymer Synthesis and Characterization of Core Crosslinked Micelles
Source: Polymers (Basel). 2017 Dec 14;9(12):710. doi: 10.3390/polym9120710 (PMC6418968; doi:10.3390/polym9120710)

Supplementary Materials

Photo Irradiation-Induced Core Crosslinked Poly(ethylene glycol)-*block*-poly(aspartic acid) Micelles: Optimization of Block Copolymer Synthesis and Characterization of Core Crosslinked Micelles

Kouichi Shiraishi [1], Shin-ichi Yusa [2], Masanori Ito [2], Keita Nakai [2], Masayuki Yokoyama [1]

[1] Medical Engineering Laboratory, Research Center for Medical Sciences, The Jikei University School of Medicine, 163-1, Kashiwashita, Kashiwa, Chiba, 277-0004, Japan

[2] Department of Applied Chemistry, Graduate School of Engineering, University of Hyogo, 2167 Shosha, Himeji, Hyogo 671-2280, Japan

Corresponding Author

∗E-mail address: masajun2093ryo@jikei.ac.jp, Tel: +81-4-7164-1111 (ext. 6710)

Alkylated chalcone derivatives

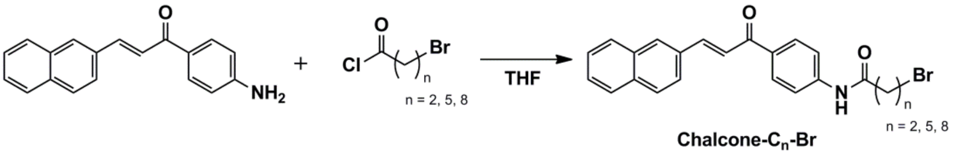

Chalcone-C$_n$-Br
1a (n=2)
1b (n=5)
1c (n=8)

Scheme S1 synthesis of alkylated chalcone derivative

Figure S1(a). ^{1}H NMR of Chal-C_8-Br in $CDCl_3$

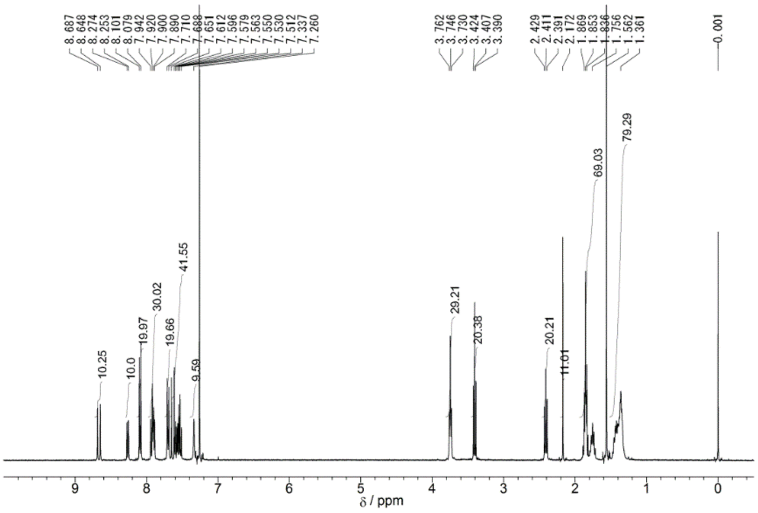

Figure S1(b). ^{1}H NMR of Chal-C_5-Br in $CDCl_3$

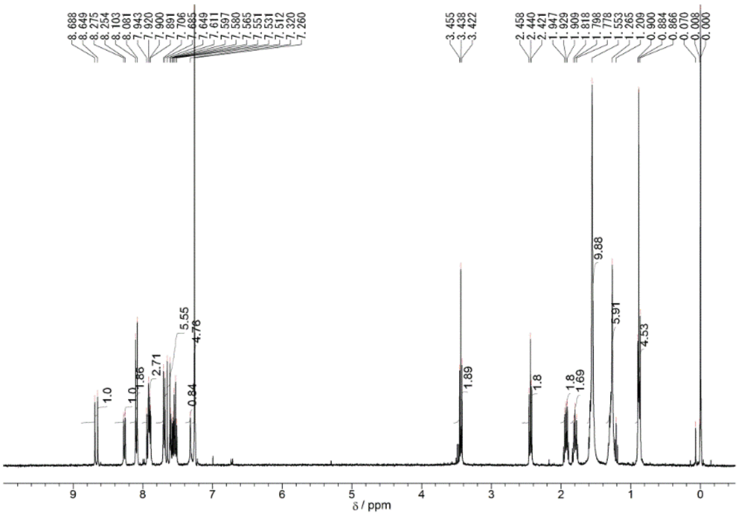

Figure S1(c). ^1H NMR of Chal-C_2-Br in $CDCl_3$

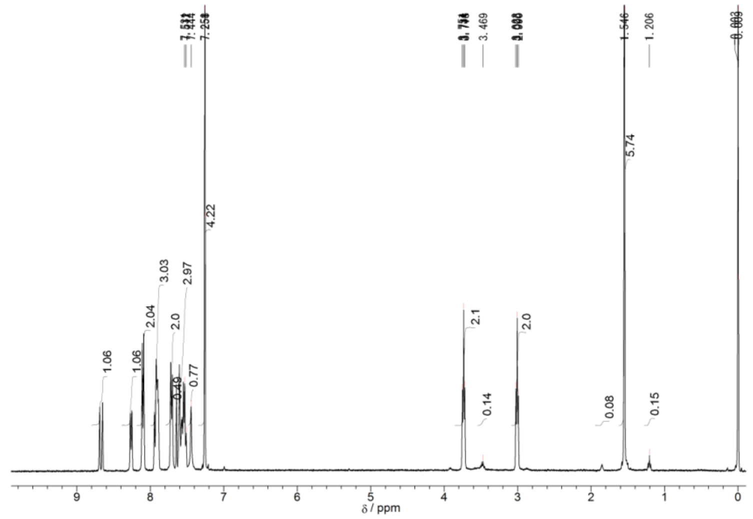

Alkaline hydrolysis of PEG-PBLA

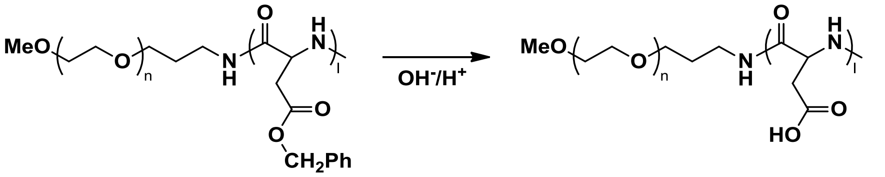

Scheme S2

Figure S1(d). ^1H NMR of PEG-P(Asp) in D_2O+NaOD (pH>10)

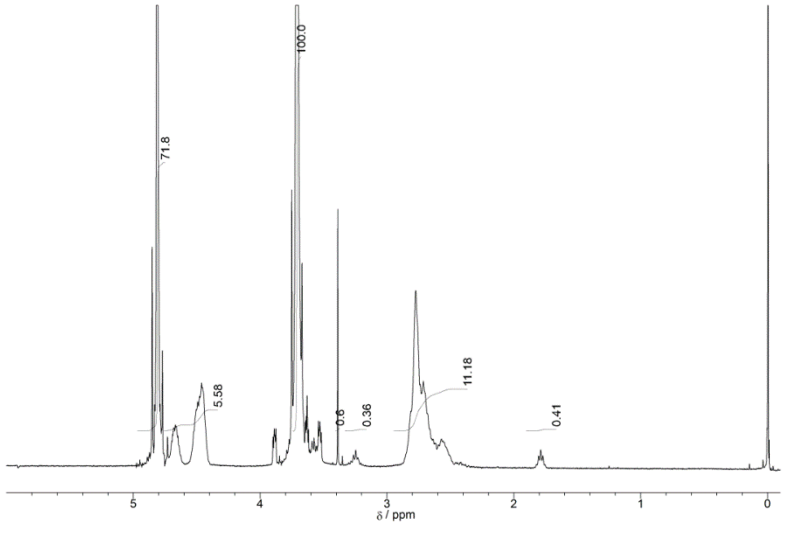

Esterification of PEG-P(Asp) with 3-bromo-N-butyl-propanamide(3-BNBPA)

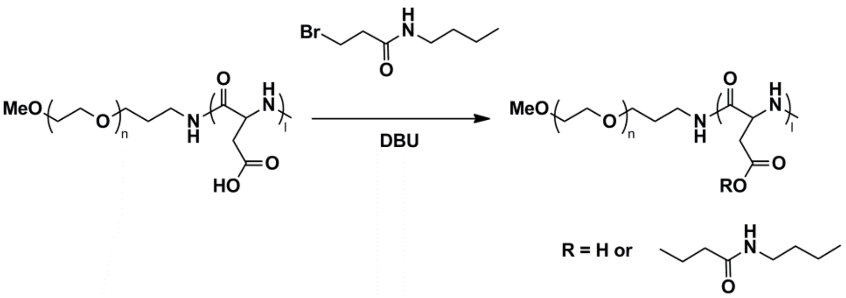

Table S1 Reaction condition of *N*-butyl-propanamide (NBPA) introduction

Polymer mg/Asp mmol	DBU mg/mmol	3-BNBPA mg/mmol	Yield /mg	Esterification yield/%*
100.9 /0.32	58.5 /0.38	131.5 /0.63	78.3	24

*Esterification yield was calculated by ratio between $-CH_2-$ (propanamide) and $-OC_2H_4-$ (PEG) in ^{1}H-NMR.

Figure S1(e). ^{1}H NMR of PEG-P(Asp-NBPA) in DMSO+TFA

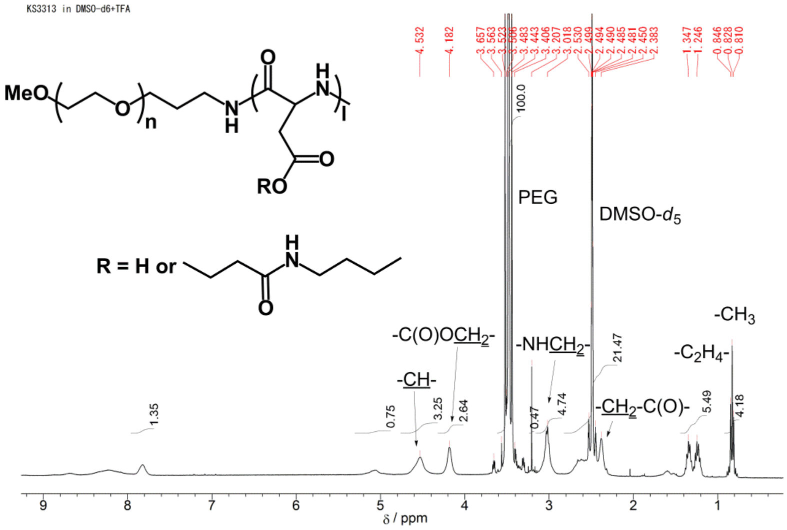

Esterification of PEG-P(Asp) with 1-iodoalkyl

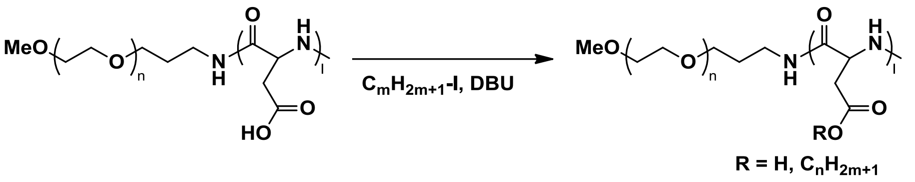

Figure S1(f). ^{1}H NMR of PEG-P(Asp-pentyl) in DMSO+TFA

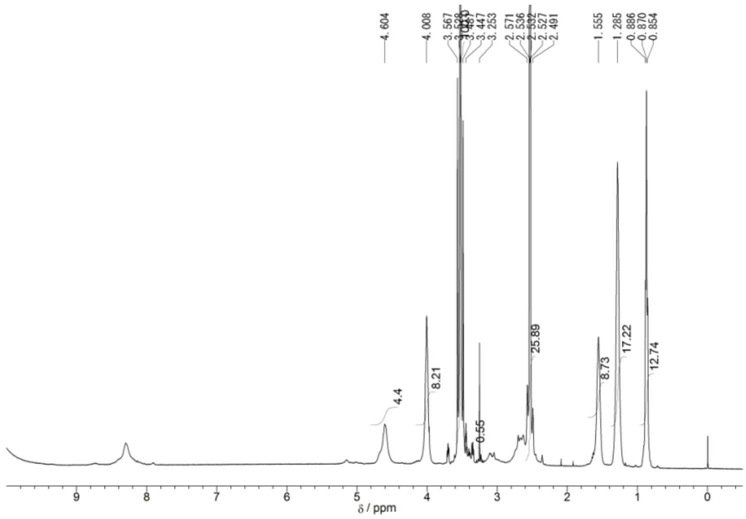

Figure S2. GPC peak area of the CCL micelle, the non-CCL micelle, and medium (saline) without the column. We used averages of peak areas of three different batches of media for estimation. Peak areas of the CCL micelle and the non-CCL micelle were estimated by the use of sample concentration and the medium volume. For example, we obtained that the non-CCL micelle's peak area was 1.59×10^6 at 1.0 mg/mL. Therefore, 88% of the non-CCL micelle was observed, whereas 12% of the non-CCL micelle was adsorbed. In contrast, the CCL micelle's peak area was 1.76×10^6 at 1.0 mg/mL (98%).

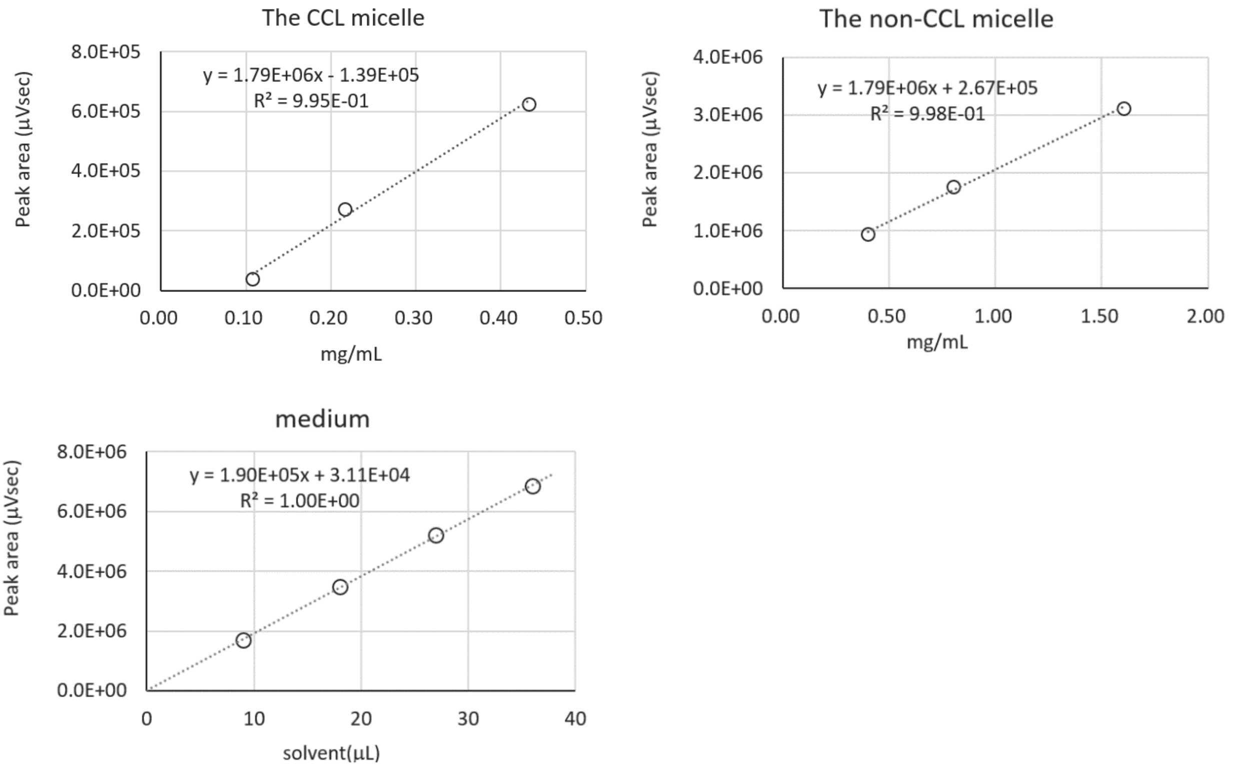

Figure S3. UV-vis spectrum of PEG-P(Asp-chal-C_2) in DMSO

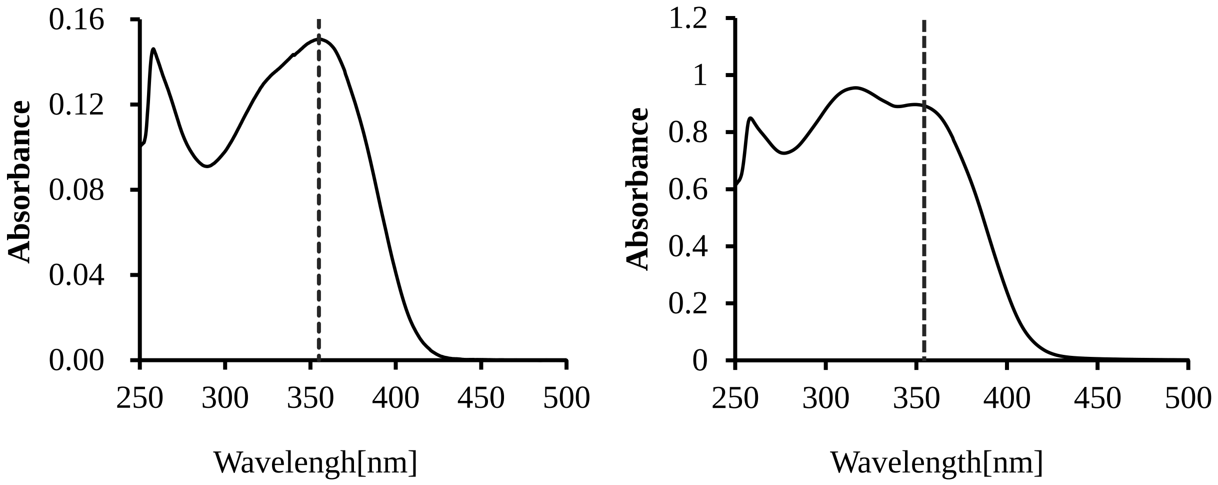

Absorption spectrum of (left) chalcone-C_2-Br and (right) PEG-P(Asp-chal-C_2) in DMSO. Absorption peak intensity at 352 nm was decrease after the reaction.

Figure S4. DLS and TEM images of aggregation form of PEG-P(Asp-nonyl-chal-C_8) (shown in run 1 in Table 3). DLS charts indicate radius of PEG-P(Asp-nonyl-chal-C_8) micelles (a) before and (b) after photo irradiation.

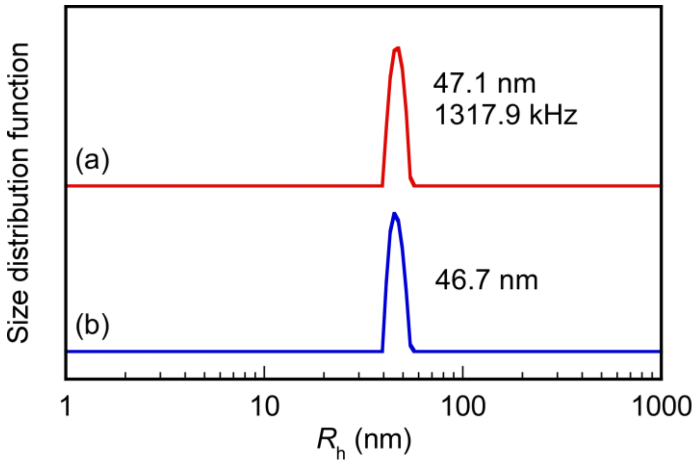

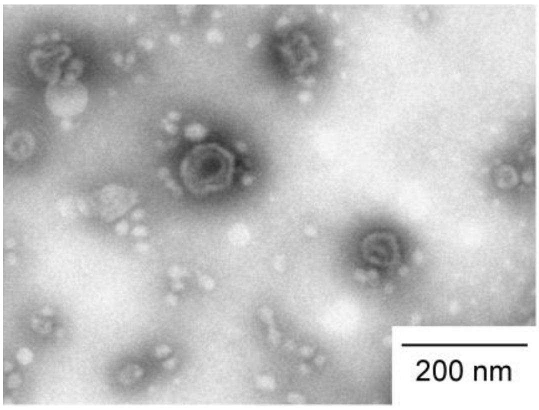

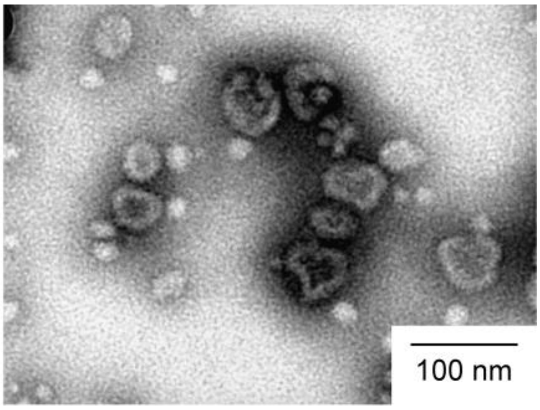

Figure S5 GPC trace of (a) non-CCL micelles and (b) CCL micelles in MeOH (containing 0.1M $LiClO_4$).

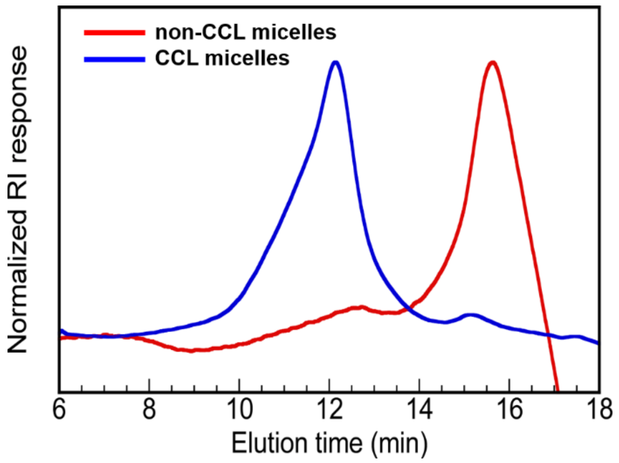

Figure S6 Fluorescence spectra of pyrene encapsulated (a) non-CCL micelles and (b) CCL micelles.
(a)

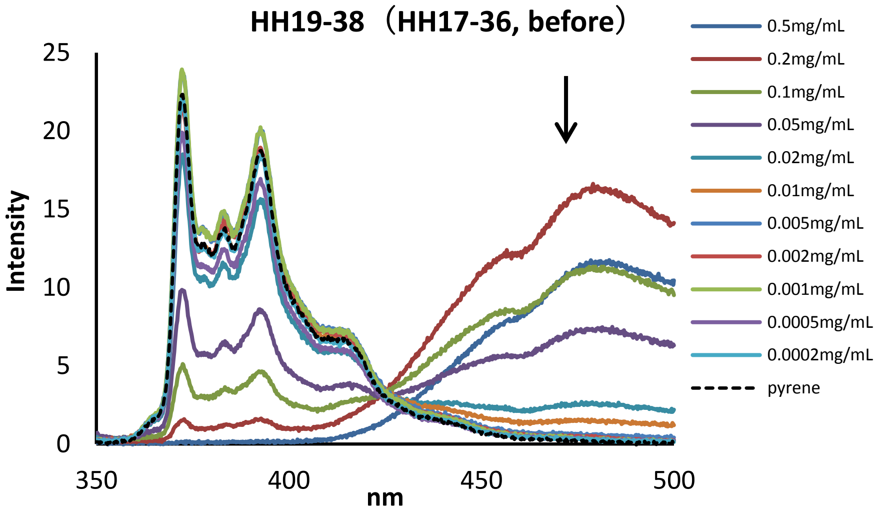

(b)

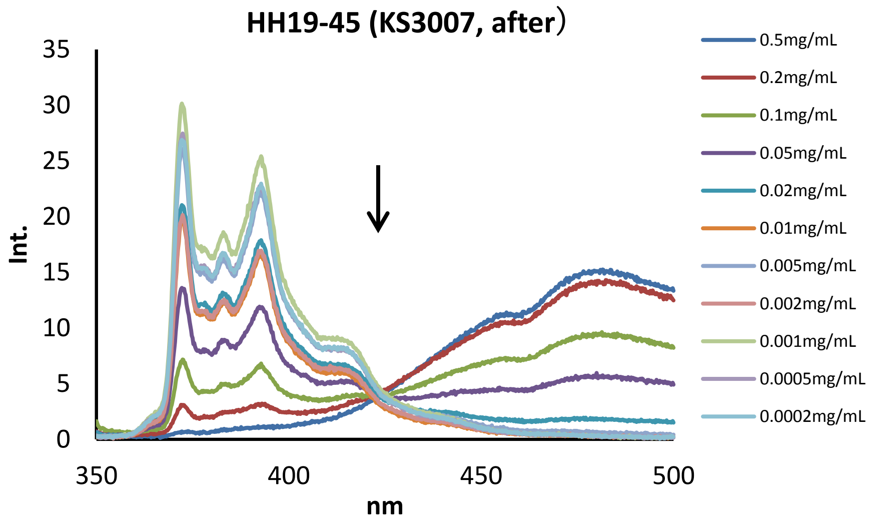

Table S2 Results of esterification in PEG-P(Asp)

Run	Chal-C_x-Br /eq*	X	DBU /eq	Chal /N	Yield /%
1	0.1	2	0.1	0.4	15.4
2	0.3	2	0.3	0.2	2.6
3	0.3	2	0.3	1.2	15.4
4	1.0	2	0.3	0.8	10.3
5	0.3	2	0.3	0.4	5.1
6	0.3	5	0.3	1.7	21.8

* Equivalent vs Asp residue.

Scheme S1 Reaction mechanism of chalcone derivative

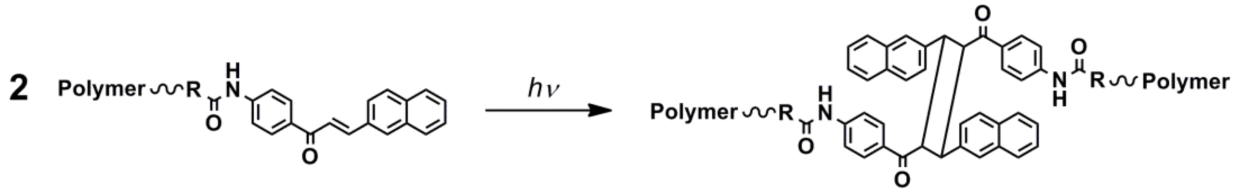

Supplement: Supplementary file 1 [file polymers-09-00710-s001.pdf]
